# High MDR‐1 expression by MAIT cells confers resistance to cytotoxic but not immunosuppressive MDR‐1 substrates

**DOI:** 10.1111/cei.13165

**Published:** 2018-09-19

**Authors:** J.R. Fergusson, J.E. Ussher, A. Kurioka, P. Klenerman, L.J. Walker

**Affiliations:** ^1^ Peter Medawar Building for Pathogen Research Oxford UK; ^2^ Department of Microbiology and Immunology University of Otago Dunedin New Zealand; ^3^ Institute of Cellular Medicine Newcastle University Newcastle upon Tyne

**Keywords:** autoimmunity, malignancy, MDR1, mucosal‐associated invariant T (MAIT) cell, transplant

## Abstract

High expression of the ATP‐binding cassette‐multi‐drug efflux protein 1 (MDR1) is a striking feature of mucosal‐associated invariant T (MAIT) cells, a prominent human innate‐like T cell subset. We demonstrate significantly higher MDR1 expression by **CD8 ^+^ CD161 ^++^ Vα7.2 ^+^** MAIT cells than the phenotypically and functionally related **CD8 ^+^ CD161 ^++^** Vα7.2‐subset and show MDR1 expression to be similarly high throughout MAIT CD4**^+^** , CD8**^+^** , double‐negative (DN) and double‐positive (DP) cell subsets. We demonstrate the MAIT cell‐predominant **CD8^+^CD161^++^** subset to uniquely and efficiently efflux the cytotoxic anthracycline daunorubicin, retain function on daunorubicin exposure and demonstrate MDR1‐dependent protection from daunorubicin‐induced apoptosis. By contrast, **CD8^+^CD161^++^Vα7.2^+^** MAIT cells were not protected from the anti‐proliferative and cytotoxic effects of the immunosuppressive MDR1 substrates tacrolimus and mycophenoic acid, although function following MAIT cell‐specific T cell receptor (TCR)‐dependent and ‐independent stimulation was preserved on ***in‐vitro*** exposure to these agents. Overall, our data further define MDR1 expression by **CD161^++^** T and MAIT cells and demonstrate the potential for high MDR1 expression by MAIT cells to confer resistance to cytotoxic MDR1 substrates ***in vivo*** *.* As our understanding of the importance of MAIT cells in human immunity and immunopathology grows, this is an important observation for clinical contexts such as the treatment of malignancy, autoimmunity and post‐transplant immunosuppression.

## Introduction

Mucosal‐associated invariant T (MAIT) cells are an abundant human innate‐like T cell subset. Representing 5–10% of peripheral T cells, MAIT cells are enriched within a number of tissues, most notably the liver, where they account for up to 50% of intrahepatic T cells [1]. MAIT cells in humans express a semi‐invariant αβ T cell receptor (TCR) and the V‐alpha chain Vα7.2 joins to restricted Jα segments (Jα33/12/20) and V‐beta chains (Vβ2 and 13.2) [2, 3]. The MAIT T cell receptor (TCR) is restricted by the highly conserved major histocompatibility complex (MHC) class I‐like molecule MR1 presenting metabolic products of the riboflavin synthesis pathways (derived from bacteria and yeasts) [4, 5, 6]. MAIT cells are characterized further by expression of the transcription factors retinoic acid‐related orphan receptor gamma T (RORγt), which drives type 17 function and phenotype [CD161, interleukin (IL)‐23R and chemokine receptor 6 (CCR6) expression] and promyelocytic leukaemia zinc finger (PLZF), related to their ‘innate’ characteristics including responsiveness to IL‐18 in synergy with IL‐12/IL‐15 and type I interferons (IFNS) [6, 7], MAIT cells are not only phenotypically distinct, but also multi‐functional – primed to respond innately to a variety of bacteria, yeast and viruses.

MAIT cells are contained entirely within the CD3^+^CD161^++^ population, and the majority of CD161^++^Vα7.2^+^ MAIT cells are either CD8^+^CD4^–^ (82.9%) or double‐negative (DN) CD8^–^CD4^–^ (12·1%), with a small minority of CD8^–^CD4^+^ and double‐positive (DP) CD8^+^CD4^+^ [8, 9]. There is little functional difference between the CD8^+^CD4^–^, CD8^–^CD4^+^ and DN CD8^–^CD4^–^ CD161^++^Vα7.2^+^ populations (although the DN population has a greater tendency to apoptosis); however, the CD8^–^CD4^+^CD161^++^Vα7.2^+^ subpopulation has reduced T helper 1 effector function but retained T helper 2 function [8]. Further to this, we have previously shown the origins of CD8^+^CD161^++^Vα7.2^+^ MAIT cells from the CD8^+^CD161^++^ subset within cord blood and demonstrated CD8^+^CD161^++^Vα7.2^+^ MAIT cells to make up 87% of the CD8^+^CD161^++^ T cell subset in adults [9].

MDR1 (or P‐glycoprotein), encoded by *ABCB1*, is the prototypical drug efflux pump that has been described to mediate multi‐drug resistance in various malignant cells [10]. It is a promiscuous transporter with broad specificity for a variety of different substrates, including those that also inhibit transport, such as cyclosporin A and verapamil [11, 12]. While much research has been invested in determining the role of MDR1 in the resistance of malignant cells to treatment, less is known about its physiological role. Expression is found in various tissues, including the gastrointestinal tract, liver, kidney, adrenal cortex, brain and testes [13] with this distribution pattern, suggesting a role in protection from xenobiotics or endogenous metabolites [10]. Expression has also been described in leucocytes [14], with a role in secretion of cytokines and cytotoxic mediators within these cells being proposed [15].

Among CD8^+^ T leucocytes MDR1 expression, the associated ability to efflux the fluorescent MDR1 substrate rhodamine 123 (Rh123) and *in‐vitro* and *in‐vivo* resistance to daunorubicin was shown initially to be restricted to a CD8^+^CD161^++^IL18Rα^++^ memory T cell subset [16], resembling but not specifically identified as MAIT cells. A subsequent study then further identified high MDR1 expression by CD4^–^CD161^++^Vα7.2^+^ T cells compared to CD4^–^CD161^+^Vα7.2^–^, CD4^–^CD161^–^Vα7.2^+^ and CD4^–^CD161^–^Vα7.2^–^ subsets, and demonstrated the ability of the CD4^–^CD161^++^Vα7.2^+^ subset alone to efflux Rh123. The same study also showed preferential survival of CD4^–^CD161^++^Vα7.2^+^ T cells in patients both during and after anthracycline‐containing chemotherapy compared to conventional memory cells on *ex‐vivo* analysis [17]. Given that MAIT cells have been shown recently to be enriched within solid organ malignancies, where they are associated with poor prognosis [18, 19, 20, 21] and identified among previously unclassified peripheral T cell lymphomas [22], further assessment of the effect of exposure to cytotoxic agents on MAIT cell survival and function is an important area to explore.

A number of immunosuppressive agents used in transplantation medicine and the treatment of autoimmunity are also substrates of MDR1 [13], and reports indicate the significance of MDR1 expressing mononuclear cells in both transplant rejection [23, 24] and treatment‐resistant autoimmunity [25, 26, 27]. MAIT cells are inherently cross‐reactive due to their restriction by the highly evolutionary conserved MR1 allowing for alloactivation through the presentation of bacterial‐derived ligands. Bystander TCR‐independent cytokine‐mediated activation of MAIT cells may also occur in the context of inflammation and the production of MAIT‐activating cytokines such as IL‐12 and IL‐18. Preferential survival of MAIT cells in the context of immunosuppression might have both beneficial and deleterious effects; on one hand, allowing them to play an important role in maintenance of immunity and on the other hand as mediators of rejection in transplantation or of treatment resistant disease in autoimmunity.

To date, published data on the role of MDR1 on MAIT cells and MAIT‐containing T cell subsets are limited to *in‐vitro* studies of anthracyline resistance of the CD161^++^IL18R^+^MDR1^+^ T cell subset [16] and the specific Rh123 efflux ability of CD4^–^CD161^++^Vα7.2^+^ cells, along with *ex‐vivo* analysis demonstrating preferential survival of CD4^–^CD161^++^Vα7.2^+^ cells following anthracycline‐containing chemotherapy compared to conventional memory cells [17]. In this study we further define the expression of MDR1 on CD161^++^ and MAIT T cell subsets. We demonstrate the ability of CD8^+^CD161^++^ cells to efflux the anthracycline daunorubicin efficiently and describe the effect of *in‐vitro* exposure to daunorubicin on CD8^+^CD161^++^ T cell survival and function. Furthermore, we investigate for the first time, to our knowledge, the effects of the immunosuppressive MDR1 substrates tacrolimus, mycophenolic acid (MPA) (the active metabolite of mycyophenolate mofetil) and the corticosteroid prednisolone on MAIT cell proliferation, survival and function.

## Materials and methods

### Cells

Peripheral blood mononuclear cells (PBMC) were obtained from whole blood leucocyte cones (NHS Blood and Transplant, Watford, UK), after ethical approval by the Central Office for Research Ethics Committees (local research ethics committee Oxford: COREC), reference number COREC 04.OXA.010.

### Flow cytometry

Dead cells were excluded with the Near‐IR Dead‐Cell stain (Invitrogen, Paisley, UK). Antibodies used were: anti‐CD3 phycoerythrin‐cyanin7 (PE‐Cy7) or allophycocyanin (APC), anti‐CD8 peridinin chlorophyll (PerCP)‐Cy5.5 or eFluor 450 (eBioscience, Hatfield, UK); anti‐CD161 PE or APC, anti‐CD8 VioGreen, anti‐interferon (IFN) fluorescein isothiocyanate (FITC) (Miltenyi Biotec, Surrey, UK); anti‐V7.2 PE or FITC or PECy7, anti‐perforin Pacific Blue, anti‐CD243/MDR1 PE (Biolegend, London, UK); anti‐granzyme B AlexaFluor700, anti‐perforin FITC, anti‐IFN AlexaFluor700 (BD Biosciences, Oxford, UK) and anti‐granzyme B APC (Invitrogen). For intracellular antibody staining cells were stained with the forehead box protein 3 (FoxP3)/transcription factor staining buffer set (eBioscience, Birmingham, UK).

Data were acquired on a MACSQuant (Miltenyi Biotec) or LSRII (BD Bioscience) and analysed using FlowJo software version 9 (Treestar, Inc., Ashland, OR, USA).

### Daunorubicin efflux assay

Fresh PBMCs were loaded with 2·5 M daunorubicin hydrochloride (Sigma, Poole, UK) for 20 min at 37°C and allowed to efflux for 1 h. Loading controls were kept on ice. After efflux, cells were returned to ice and surface stained for analysis. Daunorubicin fluorescence was measured at 610/20 nm on the LSRII (BD Biosciences). Efflux was calculated as:

(GeoMFI fluorescent substrate)_Loading control_ (GeoMFI fluorescent substrate)_Efflux_

(GeoMFI fluorescent substrate)_Loading control_

### Cytotoxicity assay

PBMCs were cultured with various concentrations of daunorubicin, tacrolimus, MPA and prednisolone (Sigma) and apoptosis detected by intracellular staining for activated caspase 3 (BD Pharmingen, Wokingham, UK) or by annexin V (Miltenyi Biotec) surface staining after 24 or 48 h, respectively. Annexin V staining was performed for 15 min at room temperature in annexin V binding buffer (Miltenyi Biotec); 50 M Verapamil (Sigma) was added at the beginning of culture. Percentages expressing annexin V at each concentration were background subtracted for annexin V expression in media alone.

### Proliferation assay

PBMCs were thawed and washed extensively in phosphate‐buffered saline (PBS) before being stained with 2·5 M CellTrace^TM^ Violet (CTV) dye (Invitrogen) for 10 min at room temperature. Ice‐cold medium was added to quench the reaction, and cells kept on ice for 5 min. Cells were then washed three times in medium, transferring cells into new tubes between each wash. Finally, cells were incubated at 37°C for 5 min, to allow excess dye to diffuse out. Cells were then resuspended at 2 × 10^6^/ml in RPMI‐1640 medium (gibco, ThermoFisher Scientific, Waltham, MA, USA), 10% normal human serum (Sigma), 2 mM L‐glutamine (gibco) and ×1 penicillin–streptomycin solution (gibco) with anti‐CD3/2/28 activation beads (Miltenyi Biotec), as per the manufacturer’s instructions, and cultured in 48‐well flat‐bottomed plates with or without immunosuppressants tacrolimus, prednisolone or MPA (Sigma). After 4 days, cells were split and either surface‐stained for analysis of CTV dilution or assessed for functional activity.

### Tamm–Horsfall protein (THP)‐1/*Esherichia coli* stimulation assay

THP‐1 cells [European Collection of Authenticated Cell Cultures (ECACC), Salisbury, UK] were incubated overnight with paraformaldehyde‐fixed *E. coli* (DH5; Invitrogen) at 25 bacteria per cell. Following culture with immunosuppressant drugs tacrolimus, prednisolone or MPA, PBMC were washed extensively then added to washed THP1s for a 5‐h co‐culture, with 3 g/ml brefeldin A (eBioscience) added after 1 h. IFN production was assayed by intracellular cytokine staining.

### IL‐12/IL‐18 stimulation assay

Following culture with immunosuppressant drugs tacrolimus, MPA or prednisolone, PBMC were incubated with IL‐12 (Miltenyi Biotec) and IL‐18 (R&D Systems, Abingdon, UK), each at 50 ng/ml overnight; 3 μg/ml brefeldin A (eBioscience) was added for the final 4 h of incubation and IFN‐γ production assayed by intracellular cytokine staining.

### CD3/CD28 bead stimulation assay

For activation through the TCR, PBMC were cultured with anti‐biotin beads coated with anti‐CD2‐biotin, anti‐CD3‐biotin and anti‐CD28‐biotin at 10 μg/ml at a bead‐to‐cell ratio of 1 : 2, according to the T cell Activation/Expansion kit (Miltenyi Biotec) instructions. IFN‐γ production was assayed after 24 h by intracellular cytokine staining.

### Statistical analysis

Statistical analysis, as stated in the figure legends, was performed using Prism version 6 software (GraphPad, San Diego, CA, USA). Data are represented as mean ± standard error of the mean (s.e.m.); ****P < 0·0001, ***P < 0·001, **P < 0·01, *P < 0·05, n.s. = not significant, as stated in the figure legends.

## Results

### MDR1 expression is similar among MAIT cell subsets and MAIT cells express the highest levels of MDR1 among CD8^+^ T cells

**Figure 1:**
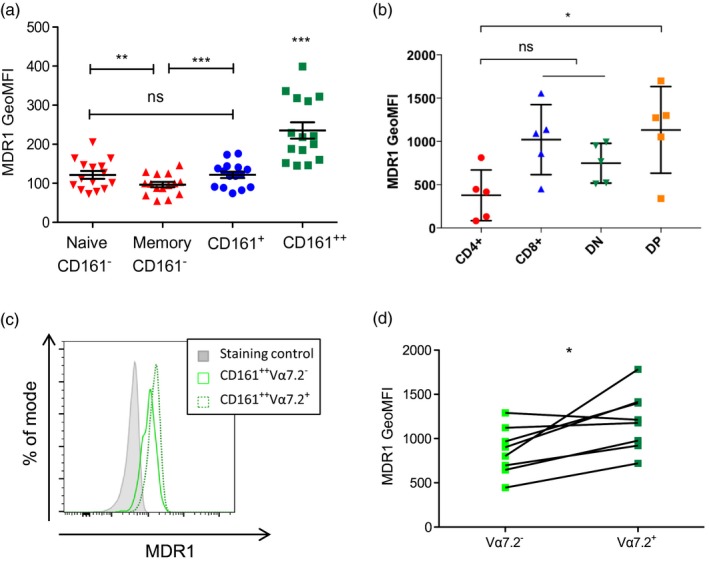
Mucosal‐associated invariant T (MAIT) cells express high levels of multi‐drug resistance protein 1 (MDR1). (a) Cumulative data showing geometric mean fluorescent intensity (GeoMFI) of MDR1 by each subset including naive (CCR7^+^CD45RA^+^) and memory (excluding CCR7^+^CD45RA^+^) CD161^–^CD8^+^ (red), CD161^+^CD8^+^ (blue) and CD161^++^CD8^+^ (green) subsets (*n* = 15). ***P < 0·001; **P < 0018301; n.s. = not significant by one‐way analysis of variance (anova) with Tukey’s multiple comparisons test. Data are represented as mean ± standard error of the mean (s.e.m.). (b) Cumulative data showing GeoMFI of MDR1 by each CD3^+^CD161^++^Vα7.2^+^ subset including CD4^+^ (red), CD8^+^ (blue), double‐negative (DN)/CD4^–^CD8^–^ (green) and double‐positive (DP)/CD4^+^CD8^+^ subsets (orange) (*n* = 5). *P < 0.05; n.s. = non‐significant by Kruskal–Wallis with Dunn’s multiple comparison test. (c) Representative flow cytometry data of MDR1 expression by CD161^++^CD8^+^ subsets, either Vα7.2^+^ (green dotted line) or Vα7.2^–^ (green solid line), compared to the fluorescence minus one (staining control) sample (grey). (d) Paired data for geometric mean fluorescent intensity (GeoMFI) of MDR1 by CD161^++^CD8^+^Vα7.2^+^ and CD161^++^CD8^+^Vα7.2^–^ T cells. *P < 0·05 by paired *t*‐test.

CD161^++^CD8^+^IL‐18R^+^ T cells [16], and specifically CD4‐CD161^++^Va7.2^+^ MAIT cells [17], have been described to express high levels of the multi‐drug efflux pump MDR1. Here we demonstrate naive CD161^–^CD8^+^ cells to express similar levels of MDR1 to CD161^+^ memory CD8^+^ T cells, and both subsets to express higher levels than their CD161^+^CD8^+^ memory counterparts. Furthermore, CD161^++^CD8^+^ T cells, the majority of which were MAIT cells [9], expressed significantly higher levels of MDR1 than all other CD8^+^ T cell populations, confirming previous studies (Fig. 1a) (gating strategies are shown in Supporting information, Fig. S1a).

Following from our work looking at differential phenotypes and functions of MAIT cell subsets [8], we further defined MDR1 expression throughout MAIT subsets based on CD4 and CD8 co‐receptor expression. CD4^+^ MAITs express significantly less MDR1 compared to the DP subset, but otherwise no statistically significant difference between subsets was observed (Fig. 1b). (see Supporting information, Fig. S1b for gating strategy and Supporting information Fig. S1c for MAIT subset distribution within our data set).

We have shown previously that the effector memory CD8^+^CD161^++^Vα7.2^+^ MAIT cell population is derived from a pool of naive CD8^+^CD161^++^ T cells identified in cord blood [9], and for CD8^+^CD161^++^Vα7.2^‐^ T cells to share many important phenotypical and functional characteristics with MAIT cells, including expression of the transcription factors PLZF, RORγt and TCR‐independent activation by IL‐12/IL‐18 [28]. We therefore compared MDR1 expression on the CD8^+^CD161^++^Vα7.2^+^ and CD8^+^CD161^++^Vα7.2^–^ subsets and demonstrated similarly high expression of MDR1 by both CD8^+^CD161^++^Vα7.2^+^ MAIT cells and the CD8^+^CD161^++^Vα7.2^–^ subset, although MDR1 expression was significantly higher on CD8^+^CD161^++^Vα7.2^+^ MAIT cells compared to the CD8^+^CD161^++^Vα7.2^–^ subset (Fig. 1c).

### MAIT cells are protected from cytotoxicity during exposure to daunorubicin

**Figure 2:**
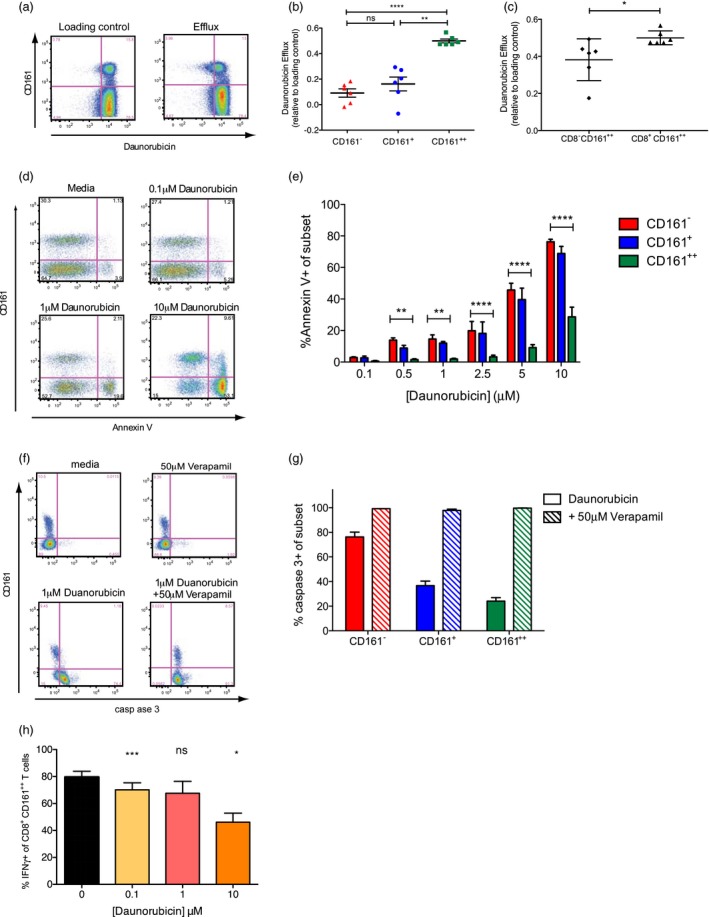
Mucosal‐associated invariant T (MAIT) cells are protected from cytotoxicity during exposure to daunorubicin. (a) Representative flow cytometry data of peripheral blood mononuclear cells (PBMC) loaded with daunorubicin and cultured on ice (loading control; left) or at 37°C (efflux; right) and gated on CD8^+^CD3^+^ lymphocytes. (b) Cumulative data for daunorubicin efflux by each CD8^+^ T cell subset calculated as per materials and methods (*n* = 6). ***P < 0·001; **P < 0·01; *P < 0·05; n.s. = not significant by one‐way analysis of variance (anova) with Tukey’s multiple comparisons test. Data are represented as mean ± standard error of the mean (s.e.m.). (c) Cumulative data for duanorubicin efflux by CD8^+^CD161^++^ and CD8^–^CD161^++^ subsets calculated as per materials and methods (*n* = 6). *P < 0·05 by Mann–Whitney test. Data are represented as mean ± s.e.m. (d) Representative flow cytometry plots of annexin V staining of PBMC cultured for 48 h in media alone or with various concentrations of daunorubicin, and gated on CD8^+^CD3^+^ lymphocytes. (e) Cumulative data for percentage of CD161^–^ (red), CD161^+^ (blue) and CD161^++^ (green) CD8^+^ T cells stained by annexin V after culture for 48 h with various concentrations of daunorubicin. ***P < 0.001; **P < 0.01; *P < 0.05; n.s. = not significant by one‐way anova with Tukey’s multiple comparisons test; all other comparisons were not significant. Data are represented as mean ± s.e.m. (f) Representative flow cytometry plots of caspase 3 expression in CD8^+^CD3^+^ lymphocytes from PBMC after culture with media alone or 1 μM daunorubicin, with or without the MDR1 inhibitor verapamil (50 μM), for 48 h. Cells cultured with verapamil alone are also shown. (g) Cumulative data for percentage of each subset, expressing caspase 3 after culture with daunorubicin alone (block colour) or with verapamil (hatched bars) (*n* = 5). Data are represented as mean ± s.e.m. (h) Cumulative data for percentage of MAIT cells expressing interferon (IFN)‐γ in response to *Escherichia coli*‐loaded Tamm–Horsfall protein (THP)1 cells after 48 h culture in the presence of various concentrations of daunorubicin (*n* = 6). ***P < 0·001, *P < 0·05, n.s. = not significant by one‐way anova with Dunnett’s multiple comparison test, compared to untreated cells. Data are represented as mean ± standard error of the mean (s.e.m.).

We have previously proved the functional activity of MDR1 on CD8^+^CD161^++^ cells using the fluorescent substrate Rh123 and demonstrated inhibition by the MDR1 inhibitors cyclosporin A and verapamil [29]. While efflux was not as marked as for Rh123, the CD8^+^CD161^++^ T cells were able to efflux the fluorescent anthracycline daunorubicin significantly more efficiently than other CD8^+^ T cell subsets, both CD8^+^CD161^+^ (*P* < 0·01) and CD8^+^CD161^–^ (*P* < 0·001) (Fig. 2a,b). Further to this, we demonstrate that the CD8^+^CD161^++^ subset is able to efflux daunorubicin significantly more efficiently than the CD8^–^CD161^++^ subset (Fig. 2c), which may be accounted for by different levels of MDR1 expression (see Fig. 1d) and may add to the greater apoptotic tendency of the DN MAIT cell subset [8].

It has been demonstrated previously that CD4^–^CD161^++^Vα7.2^+^ MAIT cells have a superior ability to survive anthracycline‐containing chemotherapy in patients undergoing treatment for breast cancer compared to other T cell subsets [17]. We determined the ability of CD8^+^CD161^++^ cells (which are predominantly Vα7.2^+^ MAIT cells) to survive exposure to anthracycline preferentially by culture of PBMCs with daunorubicin for 48 h, with apoptosis evaluated by annexin V staining. When CD8^+^ T cells were gated based on the level of CD161 expression, CD161^++^CD8^+^ T cells were found to be significantly more resistant to daunorubicin‐induced apoptosis at concentrations of 0·5–10 μM/l compared to the other CD8^+^ T cell subsets (Fig. 2d,e). Such protection was attributable to their expression of MDR1, as resistance was abrogated by addition of the MDR1 inhibitor verapamil, determined by staining for activated caspase 3, an additional marker of apoptosis (Fig. 2f,g).

In addition to resisting cytotoxic effects, we next investigated whether CD8^+^CD161^++^ cells remained functional after exposure to daunorubicin by co‐culture of daunorubicin‐exposed PBMCs with *Escherichia coli*‐pulsed THP1s, shown previously to specifically activate the MAIT cell subset [6, 27]. While CD8^+^CD161^++^ cells retained the ability to respond, the percentage of live cells producing IFN‐γ in response to *E. coli* was reduced upon culture with daunorubicin, particularly at a concentration of 10 μM, where the response was reduced significantly by more than 40% (mean 79·65 *versus* 46·10%). A smaller reduction in function was also observed at lower concentrations (Fig. 2h).

### MAIT cells are susceptible to immunosuppressant MDR1 substrates but remain functional after in‐vitro exposure

The ability of CD161^++^CD8^+^ T cells to survive culture with chemotherapeutic agents through drug efflux suggests that they may also possess the ability to efflux, and hence be resistant to, other pharmacological MDR1 substrates. We therefore investigated the ability of CD8^+^CD161^++^Vα7.2^+^ MAIT cells to withstand the effects of the immunosuppressant MDR1 substrates tacrolimus, MPA and prednisolone.

**Figure 3:**
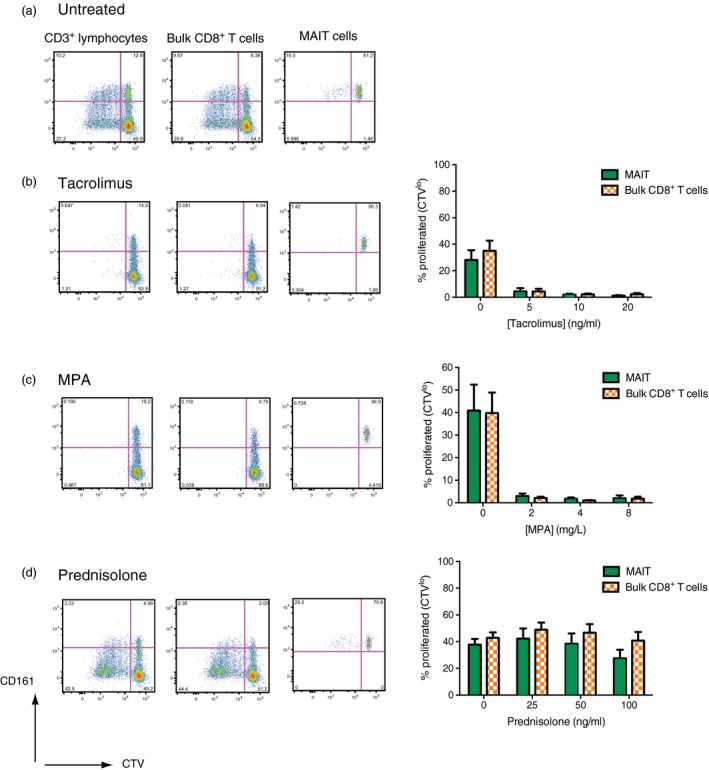
Mucosal‐associated invariant T (MAIT) cells are susceptible to the anti‐proliferative effects of tacrolimus and mycophenolic acid (MPA). (a) Representative flow cytometry plots showing CellTrace^TM^ Violet (CTV) dilution upon proliferation of CD3^+^ lymphocytes, non‐MAIT bulk CD8^+^ T cells (CD3^+^CD8^+^ lymphocytes, excluding CD161^++^Vα7.2^+^) or MAIT cells (CD3^+^CD8^+^CD161^++^Vα7.2^+^) after activation with anti‐CD3/CD2/CD28 beads for 4 days. Representative flow cytometry plots and cumulative data for the percentage of either MAIT (green) or non‐MAIT bulk CD8^+^ T cells (orange hatch) proliferated (CTV^lo^) after 4 days’ culture with anti‐CD3/CD2/CD28 beads are shown for cells cultured in the presence of either tacrolimus (b), MPA (c) and prednisolone (d). Representative flow cytometry plots are shown for the middle concentration of each drug, namely 10 ng/ml tacrolimus, MPA 4 mg/ml and prednisolone 50 ng/ml. Gates were set according to unstimulated controls (*n* = 6).

We first examined the effect of tacrolimus, MPA and prednisolone on CD8^+^CD161^++^Vα7.2^+^ MAIT cell proliferation, assayed by CTV dilution, upon anti‐CD3/CD2/CD28 stimulation for 4 days (Fig. 3a) in the presence or absence of physiological concentrations of tacrolimus (Fig. 3b), MPA (Fig. 3c) or prednisolone (Fig. 3d). The percentage of cells that had proliferated was determined by identifying cells that had lost CTV fluorescence (CVT^lo^), representing daughter cells. In the absence of drugs, both CD8^+^CD161^++^Vα7.2^+^ MAIT cells and bulk (non‐MAIT) CD8^+^ T cells proliferated in response to TCR stimulation (Fig. 3a). However, both subsets were inhibited similarly by tacrolimus and MPA at all concentrations of drugs used in the assay (Fig. 3b,c). By contrast, both CD8^+^CD161^++^Vα7.2^+^ MAIT and bulk (non‐MAIT) CD8^+^ T cell proliferation remained uninhibited in the presence of prednisolone at the concentrations used (Fig. 3d). Levels of MAIT cell apoptosis, as measured by annexin V staining, following 4 days’ culture with various concentrations of tacrolimus, MPA and prednisolone, was not significantly different from cells cultured in media alone (Supporting information Fig. S2a).

We next explored the effect of co‐culture with tacrolimus, MPA and prednisolone on CD8^+^CD161^++^Vα7.2^+^ MAIT cell TCR‐dependent and ‐independent functions. MAIT cells retained their ability to produce IFN‐γ on stimulation by both *E. coli‐*loaded THP1 cells at similar levels to untreated cells (Supporting information, Fig. S3a). Response was affected non‐significantly by a combination of all three drugs (Supporting information, Fig. S2b).

MAIT cells have also been described to respond to the combination of IL‐12 and IL‐18 in a TCR‐independent manner [7]. This response was also maintained after exposure to all three drugs, among all drug concentrations tested (Supporting information, Fig. S3b). As both these assays activate MAIT cells specifically, no direct comparison with the effect on function of non‐MAIT T cells could be made in these experiments.

**Figure 4:**
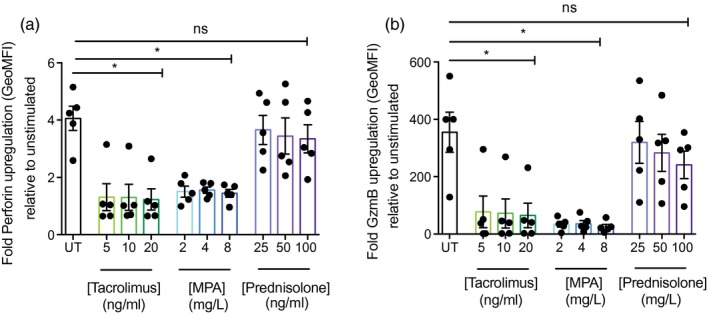
Up‐regulation of cytotoxic mediators is inhibited in mucosal‐associated invariant T (MAIT) cells exposed to tacrolimus and mycophenolic acid (MPA). Cumulative data for fold up‐regulation of perforin (a) and granzyme B (b) by CD8^+^CD161^++^Vα7.2^+^ MAIT cells relative to unstimulated controls after stimulation with anti‐CD3/CD2/CD28 beads for 24 h, following 4 days culture with various concentrations of tacrolimus, MPA or prednisolone (*n* = 5). *P < 0·05; n.s. = not significant by one‐way analysis of variance (anova) with Dunnett’s multiple comparison test, compared to untreated cells. Data are represented as mean ± standard error of the mean (s.e.m.).

In the resting state, MAIT cells lack granzyme B and express low levels of perforin, but have been shown to up‐regulate these molecules upon activation to enable cytotoxicity against target cells [30]. We therefore explored the effect of 4 days’ pre‐culture with tacrolimus, MPA and prednisolone on up‐regulation of perforin and granzyme B by CD8^+^CD161^++^Vα7.2^+^ MAIT cells following stimulation with anti‐CD3/CD2/CD28 beads. Untreated cells showed substantial up‐regulation of both cytotoxic mediators after activation, which was inhibited significantly throughout all concentrations of tacrolimus and MPA tested, but not following exposure to prednisolone (Fig. 4a,b), reflecting proliferative responses (Fig. 3).

## Discussion

Interest in and evidence for the significance of MAIT cells to human immunity is building – not least in terms of their prevalence, location at key sites of pathogen entry and conserved innate multi‐functionality against bacterial, fungal and viral infections. Many clinical studies have indicated involvement of MAIT cells in the context of infection, and a role in autoimmunity and malignancy is also emerging (reviewed in [1]). Markedly high expression of MDR1 specifically by MAIT cells is demonstrated clearly by our data, with similar expression among all MAIT subsets. Their resistance to the effects of anthracyline cytotoxic MDR1 substrates is a significant observation and important to consider in the clinical contexts of solid organ and haematological malignancy. Furthermore, MAIT cell susceptibility to the anti‐proliferative and cytotoxic effects of the immunosuppressive agents tacrolimus and MPA has important implications for related research in the fields of autoimmunity and transplantation and a role in driving immunosuppression resistant disease/rejection, as hypothesized, less likely.

There are few studies of the role of MAIT cells in malignancy, but their accumulation has been demonstrated in kidney and brain cancer [19], with the degree of MAIT cell infiltration and activation correlating negatively with life expectancy in colorectal adenocarcinoma [18, 20, 21]. In addition, MAIT cells have been described to account for a significant proportion of previously unclassified peripheral T cell lymphomas [22]. Our data, in keeping with previous studies [16, 17], further demonstrate MAIT cells to preferentially survive exposure to cytotoxic MDR1 substrates. We show not only a significantly greater efflux of daunorubicin from MAIT cells compared to other cell subsets, in line with data showing efflux by effector memory CD161^++^CD8^+^ T cells [16], but also directly demonstrate MDR1‐mediated protection from apoptosis for the first time, previously correlated only with preferential survival in patients receiving chemotherapy for breast cancer over other lymphocyte subsets [17]. Furthermore, we show daunorubicin exposed MAIT cells maintain, albeit reduced, function following physiological MR1‐mediated stimulation through the MAIT cell TCR. All experiments, however, were conducted with MAIT cells exposed to daunorubicin in a quiescent state. It would be interesting to know if the same protection is afforded to cells activated prior to drug exposure.

At this stage it is difficult to interpret the negative correlations between MAIT cell infiltration, tumour biology and life expectancy. Whether infiltration by high numbers of MAIT cells is important for anti‐cancer immunity and a correlate of an aggressive tumour or whether they contribute directly to tumour growth, for example by IL‐17‐mediated recruitment of suppressive neutrophils and myeloid‐derived suppressor cells, is not known and may differ between individual malignancies. However, given our findings, further studies of the role of MAIT cells in malignancy will have important implications for cancer therapy, including better understanding of their survival and susceptibility to chemotherapeutic agents and in the potential use of MAIT cell‐targeting depletive or adoptive transfer therapy.

Potential pathogenic and protective roles of MAIT cells are described in autoimmune disease from both clinical and murine studies published in multiple sclerosis (MS), rheumatoid arthritis (RA), systemic lupus (SLE), ankylosing spondylitis, inflammatory bowel disease (IBD) and coeliac disease (reviewed in [31]). Accumulation of MAIT cells at sites of tissue inflammation, including inflammatory lesions in MS [32, 33, 34], the gut in IBD [35, 36] and synovial fluid in RA [37], suggests a pathogenic role. However, in the murine model of MS, experimental autoimmune encephalitis (EAE), in both adoptive transfer and transgenic studies, MAIT cells protected against central nervous system (CNS) inflammation and demyelination and MR1^–/–^ mice, which lack MAIT cells, suffered more severe EAE [38]. There have been no studies of MAIT cells in rejection of solid organ transplantation. There have been conflicting reports on the role of CD161^+^ and CCR6^+^ lymphocyte subsets, not formally defined as containing MAIT cells, in graft‐*versus*‐host disease (GVHD) following bone marrow or haematopoetic stem cell transplantation preceded by myeloablative conditioning [interestingly, MAIT cells do not re‐emerge post‐transplant following non‐myeloablative conditioning with anti‐CD52 therapy [39] (L. J. Walker, unpublished data) ]. These studies suggest both protective [40] and pathogenic roles, along with protection of CD161^+^CCR6^+^ cells from the anti‐proliferative effects of cyclosporin A within a mixed leucocyte reaction (MLR) model [41]. Alloactivation of MAIT cells due to their inherent cross‐reactivity via a TCR restricted to the highly evolutionarily conserved MR1 would require the presentation of bacterial antigen in the context of infection or contamination; however, bystander TCR‐independent activation may occur in an inflammatory environment and play a role in mediation and augmentation of rejection or organ dysfunction post‐transplant. For example, IL‐18 is a key activator of MAIT cells in synergy with other proinflammatory cytokines and has been implicated in both delayed liver graft function in a rat model [42] and episodes of liver transplant rejection in humans [43]. A role of MAIT cells in this setting (especially in the liver, where they are an abundant lymphocyte subset) has not been explored but would be an important area for further study.

Our data suggest that, as with daunorubicin, the MAIT cell responses to TCR‐dependent and ‐independent stimuli are preserved following exposure to the MDR1 substrates tacrolimus, MPA and prednisolone. However, MAIT cell expression of the cytotoxic molecules, granzyme B and perforin, and proliferation upon anti‐CD3/CD2/CD28 bead stimulation was inhibited similarly as in non‐MAIT cells following exposure to tacrolimus and MPA, and therefore no MDR1‐related protective effect was demonstrated. This suggests relative immunosuppression in the context of such drug treatment and may have a significant effect in terms of protective MAIT cell effector function and also unwanted autoimmunity and rejection. Prednisolone failed to inhibit proliferation of both MAIT and non‐MAIT cell subsets in this model, and MAIT cell cytotoxicity was also preserved. Previous data have demonstrated the suppressive effects of fluticasone and budesonide *in vitro* on MAIT cells using monocyte derived macrophages (MDM) co‐cultured with *Haemophilus influenzae* (HI)*.* However, in these experiments the steroids were added upon co‐culture of the MDMs with the HI and they also demonstrated down‐regulation of MR1 surface expression, indicating an immunosuppressive effect on the macrophages rather than the MAIT cells specifically [44]. In our experiments the immunosuppressive drugs were only added after overnight co‐culture of fixed *E. coli* with THP‐1 cells and so it is less likely that MR1 expression would have been affected, which may account for the differences in results. Overall, such preserved function may allow for some preservation of MAIT function in the context of immunosuppression, which may either be important in maintaining protective immunity or allow for unwanted immunosuppression‐resistant inflammation, but the observation of the Hinks study is important – as the effect of drugs on the immune system must be addressed in the context of all players and not just one cell type. However, in view of these conflicting data [41, 44], further analysis is needed using other immunosuppressive MDR1 substrates. If MAIT cells are shown to have a role in the pathogenesis of autoimmunity or transplant rejection and GVHD, these varying results would suggest that tailored therapeutic approaches may be required.

In this study we have further defined MDR1 expression by MAIT and CD161^++^ subsets and explored the effect of exposure of MAIT cells to both cytotoxic and immunosuppressive MDR1 substrates. Our data demonstrate not only that MAIT cells survive exposure to cytotoxic agents in an MDR1‐dependent manner, but also remain functional. High MDR1 expression by MAIT cells is striking among lymphocytes and further understanding of its physiological role and the implications of this within the clinical settings of malignancy, autoimmunity and transplantation is clearly required.

## Disclosure

None.

## Author contributions

J. R. F. designed the study, performed the experiments, analysed the data and drafted the manuscript. J. U. designed the study and provided technical support. A. K. provided technical support. P. K. and L. W. designed and supervised the study and drafted the manuscript.

## Supporting information

Fig. S1. (a) Gating strategy shown for analysis of fluorescence activated cell sorter (FACS) plots for Fig. 1a. (b) Gating strategy shown for analysis of FACS plots for Fig. 1b–d. (c) Collated data showing subset distribution for the CD161^++^Va7.2^+^ population.

Fig. S2. Mucosal‐associated invariant T (MAIT) cell survival in culture is not affected by exposure to tacrolimus, mycophenolic acid (MPA) or prednisolone, and function is maintained after culture with a drug combination. (a) After culture with various concentrations of tacrolimus, MPA and prednisolone for 4 days, cells were stained with annexin V. Cumulative data for percentage of MAIT cells that were annexin V‐positive (*n* = 6); n.s. = not significant by one‐way analysis of variance (anova) with Dunnett’s multiple comparison test, compared to untreated cells. Data are represented as mean ± standard error of the mean (s.e.m.). (b) After culture with a mixture of 10 ng/ml tacrolimus, 4 g/ml MPA and 50 ng/ml prednisolone for 4 days, cells were removed and incubated for 5 h with *Escherichia coli‐*loaded Tamm–Horsfall proteins (THP1s). Cumulative data for percentage of MAIT cells expressing interferon (IFN)‐γ in response (*n* = 3); n.s. = not significant by paired *t*‐test. Data are represented as mean ± standard error of the mean (s.e.m.).

Fig. S3. Mucosal‐associated invariant T (MAIT) cells retain function after exposure to tacrolimus, mycophenolic acid (MPA) and prednisolone following physiological T cell receptor (TCR)‐dependent and ‐independent stimulation. Cumulative data of percentage of MAIT cells producing interferon (IFN)‐γ after (a) 5 h co‐culture with *Escherichia coli*‐loaded Tamm–Horsfall proteins (THP1s) and (b) overnight incubation with interleukin (IL)‐12 and IL‐18 following 4 days culture in the presence of media alone or various concentrations of tacrolimus, MPA and prednisolone (*n* = 6); n.s. = not significant by one‐way analysis of variance (anova) with Dunnett’s multiple comparison test, compared to untreated cells. Data are represented as mean ± standard error of the mean (s.e.m.).
