# Supplementary material for: High MDR‐1 expression by MAIT cells confers resistance to cytotoxic but not immunosuppressive MDR‐1 substrates
Source: Clin Exp Immunol. 2018 Sep 19;194(2):180–91. doi: 10.1111/cei.13165 (PMC6194332; doi:10.1111/cei.13165)
Supplement: Supplementary file 1 — Fig. S1. (a) Gating strategy shown for analysis of fluorescence activated cell sorter (FACS) plots for Fig. 1a. (b) Gating strategy shown for analysis of FACS plots for Fig. 1b–d. (c) Collated data showing subset distribution for the CD161++Va7.2+ population. [file CEI-194-180-s001.pptx]

## Slide 1
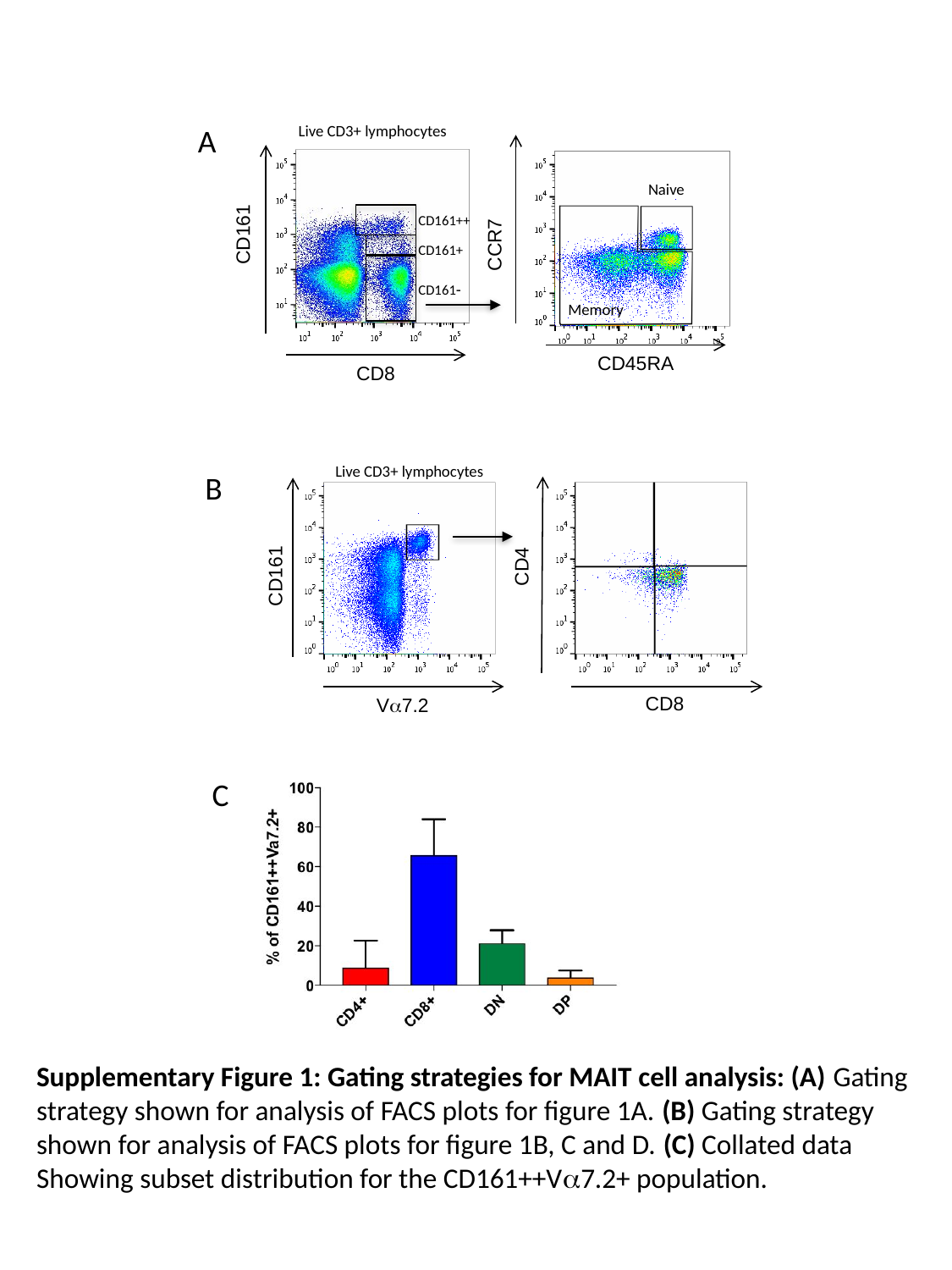

A
Live CD3+ lymphocytes
Naive
CD161++
CCR7
CD161+
CD161-
Memory
CD45RA
CD161
CD8
Live CD3+ lymphocytes
CD4
CD161
Va7.2
B
CD8
C
Supplementary Figure 1: Gating strategies for MAIT cell analysis: (A) Gating
strategy shown for analysis of FACS plots for figure 1A. (B) Gating strategy
shown for analysis of FACS plots for figure 1B, C and D. (C) Collated data
Showing subset distribution for the CD161++Va7.2+ population.
