# Supplementary material for: High MDR‐1 expression by MAIT cells confers resistance to cytotoxic but not immunosuppressive MDR‐1 substrates
Source: Clin Exp Immunol. 2018 Sep 19;194(2):180–91. doi: 10.1111/cei.13165 (PMC6194332; doi:10.1111/cei.13165)
Supplement: Supplementary file 2 — Fig. S2. Mucosal‐associated invariant T (MAIT) cell survival in culture is not affected by exposure to tacrolimus, mycophenolic acid (MPA) or prednisolone, and function is maintained after culture with a drug combination. (a) After culture with various concentrations of tacrolimus, MPA and prednisolone for 4 days, cells were stained with annexin V. Cumulative data for percentage of MAIT cells that were annexin V‐positive (n = 6); n.s. = not significant by one‐way analysis of variance (anova) with Dunnett’s multiple comparison test, compared to untreated cells. Data are represented as mean ± standard error of the mean (s.e.m.). (b) After culture with a mixture of 10 ng/ml tacrolimus, 4 g/ml MPA and 50 ng/ml prednisolone for 4 days, cells were removed and incubated for 5 h with Escherichia coli‐loaded Tamm–Horsfall proteins (THP1s). Cumulative data for percentage of MAIT cells expressing interferon (IFN)‐γ in response (n = 3); n.s. = not significant by paired t‐test. Data are represented as mean ± standard error of the mean (s.e.m.). [file CEI-194-180-s002.pptx]

## Slide 1
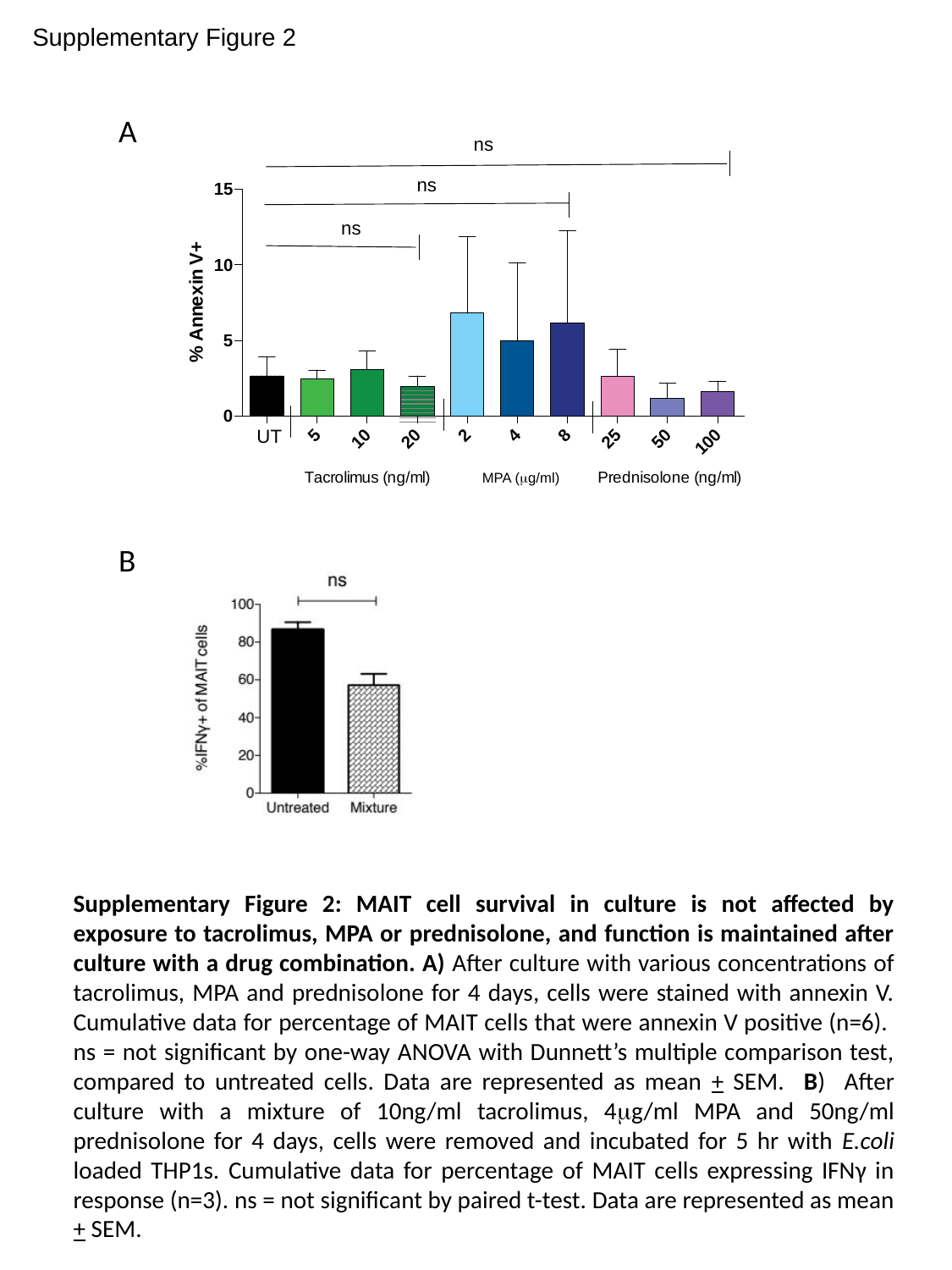

Supplementary Figure 2
A
MPA (mg/ml)
B
Supplementary Figure 2: MAIT cell survival in culture is not affected by exposure to tacrolimus, MPA or prednisolone, and function is maintained after culture with a drug combination. A) After culture with various concentrations of tacrolimus, MPA and prednisolone for 4 days, cells were stained with annexin V. Cumulative data for percentage of MAIT cells that were annexin V positive (n=6). ns = not significant by one-way ANOVA with Dunnett’s multiple comparison test, compared to untreated cells. Data are represented as mean + SEM. B) After culture with a mixture of 10ng/ml tacrolimus, 4mg/ml MPA and 50ng/ml prednisolone for 4 days, cells were removed and incubated for 5 hr with E.coli loaded THP1s. Cumulative data for percentage of MAIT cells expressing IFNγ in response (n=3). ns = not significant by paired t-test. Data are represented as mean + SEM.
