# Supplementary material for: High MDR‐1 expression by MAIT cells confers resistance to cytotoxic but not immunosuppressive MDR‐1 substrates
Source: Clin Exp Immunol. 2018 Sep 19;194(2):180–91. doi: 10.1111/cei.13165 (PMC6194332; doi:10.1111/cei.13165)
Supplement: Supplementary file 3 — Fig. S3. Mucosal‐associated invariant T (MAIT) cells retain function after exposure to tacrolimus, mycophenolic acid (MPA) and prednisolone following physiological T cell receptor (TCR)‐dependent and ‐independent stimulation. Cumulative data of percentage of MAIT cells producing interferon (IFN)‐γ after (a) 5 h co‐culture with Escherichia coli‐loaded Tamm–Horsfall proteins (THP1s) and (b) overnight incubation with interleukin (IL)‐12 and IL‐18 following 4 days culture in the presence of media alone or various concentrations of tacrolimus, MPA and prednisolone (n = 6); n.s. = not significant by one‐way analysis of variance (anova) with Dunnett’s multiple comparison test, compared to untreated cells. Data are represented as mean ± standard error of the mean (s.e.m.). [file CEI-194-180-s003.pptx]

## Slide 1
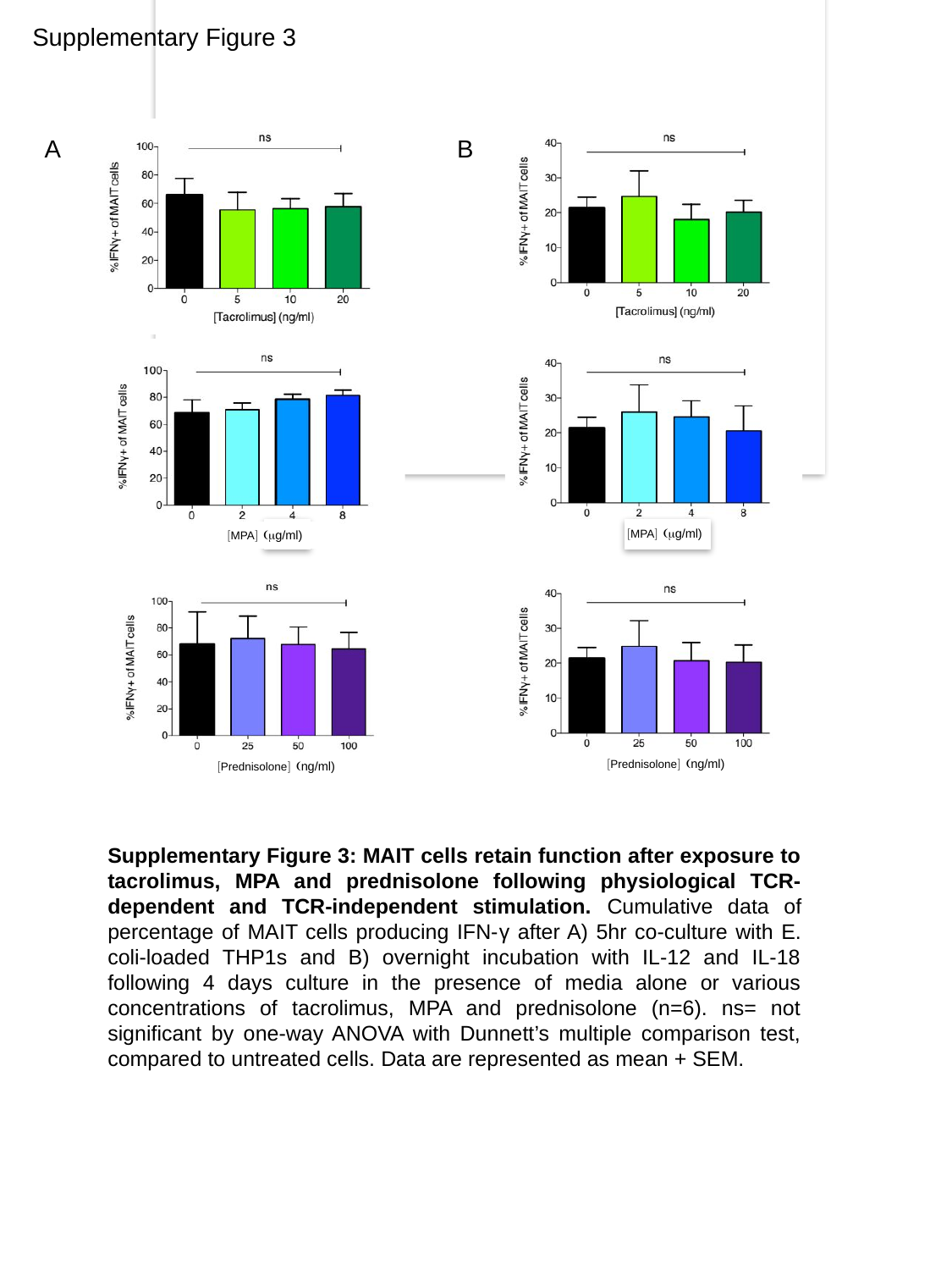

[MPA] (mg/ml)
[MPA] (mg/ml)
[Prednisolone] (ng/ml)
[Prednisolone] (ng/ml)
Supplementary Figure 3
A
B
Supplementary Figure 3: MAIT cells retain function after exposure to tacrolimus, MPA and prednisolone following physiological TCR-dependent and TCR-independent stimulation. Cumulative data of percentage of MAIT cells producing IFN-γ after A) 5hr co-culture with E. coli-loaded THP1s and B) overnight incubation with IL-12 and IL-18 following 4 days culture in the presence of media alone or various concentrations of tacrolimus, MPA and prednisolone (n=6). ns= not significant by one-way ANOVA with Dunnett’s multiple comparison test, compared to untreated cells. Data are represented as mean + SEM.
